# Survival after radiotherapy versus radical cystectomy for primary muscle‐invasive bladder cancer: A Swedish nationwide population‐based cohort study

**DOI:** 10.1002/cam4.2126

**Published:** 2019-04-01

**Authors:** Christel Häggström, Hans Garmo, Xavier de Luna, Mieke Van Hemelrijck, Karin Söderkvist, Firas Aljabery, Viveka Ströck, Abolfazl Hosseini, Truls Gårdmark, Per‐Uno Malmström, Staffan Jahnson, Fredrik Liedberg, Lars Holmberg

**Affiliations:** ^1^ Department of Surgical Sciences Uppsala University Uppsala Sweden; ^2^ Department of Biobank Research Umeå University Umeå Sweden; ^3^ Translational Oncology & Urology Research (TOUR), School of Cancer and Pharmaceutical Sciences King's College London London UK; ^4^ Regional Cancer Centre Uppsala/Örebro Uppsala Sweden; ^5^ Department of Statistics, USBE Umeå University Umeå Sweden; ^6^ Institute of Environmental Medicine Karolinska Institute Stockholm Sweden; ^7^ Department of Radiation Sciences, Oncology Umeå University Umeå Sweden; ^8^ Department of Clinical and Experimental Medicine, Division of Urology Linköping University Linköping Sweden; ^9^ Department of Urology Sahlgrenska University Hospital Gothenburg Sweden; ^10^ Department of Urology Karolinska University Hospital Stockholm Sweden; ^11^ Department of Clinical Sciences Danderyd Hospital, Karolinska Institute Stockholm Sweden; ^12^ Department of Urology Skåne University Hospital Malmö Sweden; ^13^ Department of Translational Medicine Lund University Malmö Sweden

**Keywords:** bladder cancer, muscle‐invasive, radical cystectomy, radiotherapy, urothelial carcinoma

## Abstract

**Background:**

Studies of survival comparing radical cystectomy (RC) and radiotherapy for muscle‐invasive bladder cancer have provided inconsistent results and have methodological limitations. The aim of the study was to investigate risk of death after radiotherapy as compared to RC.

**Methods:**

We selected patients with muscle‐invasive urothelial carcinoma without distant metastases, treated with radiotherapy or RC from 1997 to 2014 in the Bladder Cancer Data Base Sweden (BladderBaSe) and estimated absolute and relative risk of bladder cancer death and all‐cause death. In a group of patients, theoretically eligible for a trial comparing radiotherapy and RC, we calculated risk difference in an instrumental variable analysis. We have not investigated chemoradiotherapy as this treatment was not used in the study time period.

**Results:**

The study included 3 309 patients, of those 17% were treated with radiotherapy and 83% with RC. Patients treated with radiotherapy were older, had more advanced comorbidity, and had a higher risk of death as compared to patients treated with RC (relative risks of 1.5‐1.6). In the “trial population,” all‐cause death risk difference was 6 per 100 patients lower after radiotherapy at 5 years of follow‐up, 95% confidence interval −41 to 29.

**Conclusion(s):**

Patient selection between the treatments make it difficult to evaluate results from conventionally adjusted and propensity‐score matched survival analysis. When taking into account unmeasured confounding by instrumental variable analysis, no differences in survival was found between the treatments for a selected group of patients. Further clinical studies are needed to characterize this group of patients, which can serve as a basis for future comparison studies for treatment recommendations.

## 1 INTRODUCTION

Surgical removal of the urinary bladder, radical cystectomy (RC), is the recommended treatment for muscle‐invasive bladder cancer in most parts of the world, including Sweden,^1^ but with international variations.^2^ RC entails high risk of re‐operations, long hospital‐stays, long‐term sequelae, and even postoperative mortality.^3^, ^4^ A contemporary bladder‐sparing treatment alternative to RC is chemoradiotherapy.^5^, ^6^, ^7^, ^8^ There is no evidence from randomized controlled trials to support superiority of RC over radiotherapy with respect to survival. The largest trial to date was closed due to poor accrual,^9^ and a recent review concluded that observational studies comparing outcomes of RC to bladder‐sparing therapies had serious methodological shortcomings and inconsistent findings.^10^

The aim of this population‐based observational cohort study was to investigate the risk of bladder‐cancer specific and all‐cause death using detailed individual data on patient demographics, tumor characteristics, provided treatment, comorbidity, and follow‐up in patients with muscle‐invasive urothelial carcinoma, clinically free of metastases, and treated with radiotherapy or RC.

## 2 MATERIALS AND METHODS

### 2.1 The bladder cancer data base Sweden (BladderBaSe)

The study is based on patients diagnosed with primary muscle‐invasive disease in the Bladder Cancer Data Base Sweden (BladderBaSe). The BladderBaSe was initiated in 2015 via linkage of the Swedish National Register for Urinary Bladder Cancer (SNRUBC) to a number of health care and demographic registers.^11^ The project was approved by the Research Ethics Board at Uppsala University, Sweden.

We retrieved data on diagnosis and treatment for patients reported to the SNRUBC from 1 January 1997 to 31 December 2014. Data included date of diagnosis, diagnosing unit, morphological code, grade (G1, G2, G3, and GX), clinical T category (T0, Tis, Ta, T1, T2, T3, T4, and TX), N category (N0, N1, N2, N3, and NX), and M category (M0, M1, and MX) at diagnosis. Morphological codes were classified according to World Health Organization's International Classification of Diseases for Oncology, 3rd edition (ICD‐O/3). Clinical classification of N category was based on computed tomography and/or magnetic resonance imaging examinations of the abdomen, and N0 was defined as no evidence of regional lymph node metastases. Classification of M category was based on clinical findings and computed tomography or x‐ray examinations of the chest; and M0 was defined as no evidence of distant metastases. During 3 years in the SNRUBC, clinically node‐positive patients were only categorized as node‐positive without any further N classification. This group (less than 3% of patients with muscle‐invasive bladder cancer) was in the analysis categorized as N1 if they had grade 1, 2, or T category T2, and as N3 if they were M1, and the remaining node‐positive patients were categorized as N2. In 2013, the SNRUBC adapted the criteria for N1‐N3 to the 2009 TNM‐system.

Treatment data included reporting unit and primary treatment (RC, radiotherapy, intravesical treatment, or systemic chemotherapy), and further primary treatment registered within 6 months from date of diagnosis. The reporting unit was categorized into university, county, or district hospitals. During the investigated time period, the primary treatment with curative intent for muscle‐invasive bladder cancer was RC, with neoadjuvant chemotherapy prior to RC being gradually introduced. Patients with contraindications for RC or unwilling to accept RC were treated with radiotherapy.^1^ Radiotherapy treatment included whole pelvic radiation up to 70 Gray, with regional variations of hyper fractionated radiation during the earlier time period. We had no intention to investigate chemoradiotherapy as this treatment was not used in the study time period.

We used data from the National Patient Register on discharge diagnoses from hospital admissions up to 10 years prior to the date of bladder cancer diagnosis to calculate the Charlson Comorbidity Index (CCI), which was categorized into four groups: no comorbidity, 1, 2, and ≥ 3 comorbidities. Data on educational level were retrieved from the Longitudinal Integration Database for Health Insurance and Labor Market Studies at Statistics Sweden and categorized into three groups: ≤9 years, 10‐12 years, and ≥ 13 years of education, corresponding to low, intermediate, and high education level. To detect salvage cystectomies after radiotherapy, we retrieved data of bladder cancer (International Classification of Diseases, 10th revision [ICD‐10] code C67) and RC (procedure code KCC) or complete pelvic evisceration (procedure code LCE) from the National Patient Register. Date and cause of death were obtained from the Cause of Death Register and death from bladder cancer was defined as ICD‐10 code C67 as underlying death cause.

### 2.2 Selection of study population

Patients with primary muscle‐invasive urothelial carcinoma in the bladder without metastases (clinical T category ≥ 2, N0 and M0), treated with RC or radiotherapy were selected for the current study. Prior to selection of the study population, and in order to account for possible differences in the diagnostic workup between the groups, we imputed data on grade, N and M category for patients with clinical T category ≥ 2 and stage missing or categorized as X for grade, N or M category. The multiple imputations were based on predicted mean matching with five imputed datasets.^12^ Data on gender, age at diagnosis, date of diagnosis, health‐care region, educational level, marital status, CCI, size of reporting unit, clinical T category at diagnosis, follow‐up time, decision of further primary treatment, RC, radiotherapy, intravesical treatment, systemic chemotherapy, bladder cancer death, and other cause of death were used as independent variables for the imputation.

To provide an analysis that mimics results from a randomized controlled trial,^13^ we created a group of patients comparable to those eligible for a recent trial comparing radiotherapy and RC.^14^ For this “trial population,” we selected patients who were 60‐80 years of age at diagnosis, had clinical T category 2 or 3, and CCI less than 3 from the study population described above.

### 2.3 Statistical analysis

Starting date of the study was the date of diagnosis, and last date of the study was date of death, emigration, or 31 December 2014, whichever happened first. Time in years from diagnosis was used as timescale in all analyses.

Absolute risk of bladder cancer death and other causes of death were calculated by cumulative incidence functions using competing risk analysis.^15^ In these models, bladder cancer death was used as the main endpoint and other causes of death as a competing endpoint. Relative risk (hazard ratios) of bladder cancer death and all‐cause death were assessed by Cox proportional hazard models using RC as reference category, and adjusted for age at diagnosis (continuous), calendar year at diagnosis (1997‐2005 and 2006‐2014), education level, gender, marital status, comorbidity, health‐care region, reporting unit, grade, systemic chemotherapy, and T category. The assumption of proportional hazard was tested by adding an interaction term for treatment with time in a separate Cox model.^16^ If deviations from the proportional hazards assumption were found, hazard ratios at specific points in time were calculated.

In the “trial population,” we calculated propensity‐score matched relative risks and risk differences using instrumental variable analysis, details, and assumptions of these methods are described in Methods S1. We used treatment preference in the treating units to define the instrument, which was quantified by the proportion (0‐1) of RCs among previous patients at the same unit, whilst excluding the first patient in each unit from the analysis. All analyses were performed with STATA MP/2 version 14 (StataCorp LP, College Station, Texas).

## 3 RESULTS

**Figure 1:**
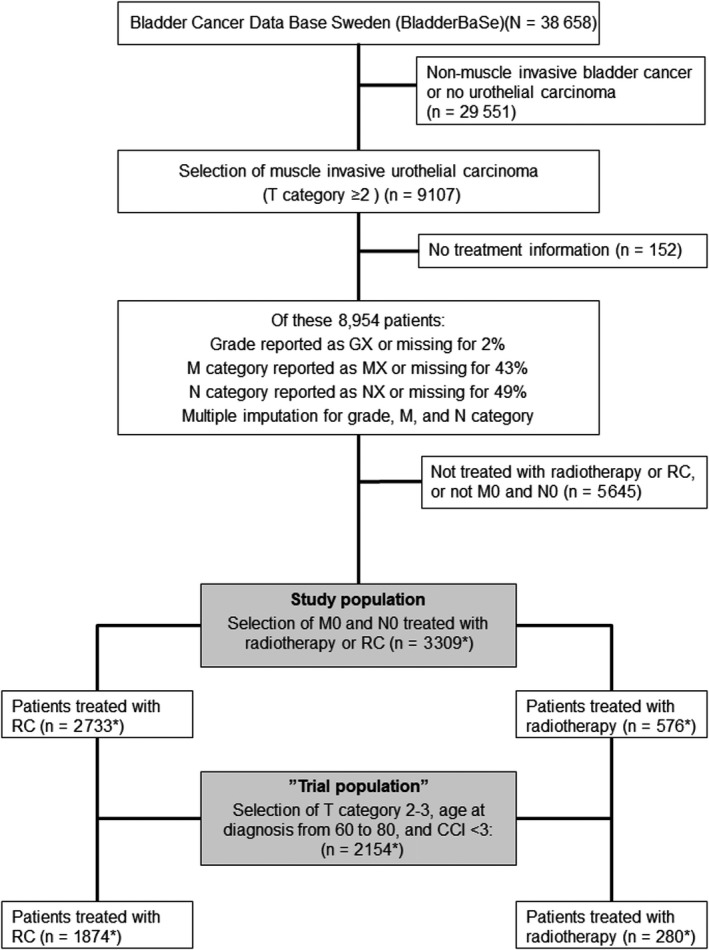
Flowchart that describes the selection of patients for the study from the Bladder Cancer Data Base Sweden (BladderBaSe), 1997 to 2014. *Pooled estimate of five imputations

From the 8 954 patients with primary muscle‐invasive urothelial carcinoma in BladderBaSe, we identified 3 309 patients with no metastases whom were treated with either radiotherapy or RC (as presented in Figure 1). Mean age at the start of study was 70 (SD = 9, median = 71, range (36, 92)), and 17% of the patients were treated with radiotherapy and 83% with RC (Table 1). Baseline data indicated that the two treatment groups had a similar distribution with respect to gender, clinical T category, and grade, but large differences in age at diagnosis and comorbidity. Systemic chemotherapy was given to 4% of patients treated with radiotherapy and 19% of patients treated with RC, and salvage cystectomy was recorded for 3% of patients treated with radiotherapy. During a mean follow‐up time of 4 years (SD = 4, median = 2, range (0, 18)), 39% of the study participants died of bladder cancer and 19% died of other causes.

**Table 1:** Baseline characteristics of the entire study population with respect to actual treatment

Covariate	Categories	Radical Cystectomy	Radiotherapy
		N = 2 733*	N = 576*
Gender	Men	75%	76%
	Women	25%	24%
Age	Below 75 years	75%	31%
	75 years and above	25%	69%
Calendar year of diagnosis	1997‐2005	42%	50%
	2006‐2014	58%	50%
Clinical T category	T2	77%	75%
	T3	18%	18%
	T4	6%	6%
Grade**	G2	12%	12%
	G3	88%	88%
CCI	0	71%	49%
	1	14%	23%
	2	10%	15%
	3+	5%	13%
Education level ***	Low	45%	55%
	Medium	37%	33%
	High	18%	12%
Marital status	Married	60%	54%
	Divorced/Widowed	28%	38%
	Unmarried	12%	8%
Health‐care region	Stockholm	16%	9%
	Southern	23%	22%
	Southeast	11%	19%
	Uppsala/Örebro	23%	27%
	Western	19%	4%
	Northern	9%	19%
Reporting unit category	University hospitals	45%	36%
	County hospitals	41%	43%
	District hospitals	14%	20%
Systemic chemotherapy	Yes	19%	4%
	No/NA	81%	96%
Neoadjuvant chemotherapy****	Yes	13%	2%
	No/NA	87%	98%
Adjuvant chemotherapy****	Yes	5%	1%
	No/NA	95%	99%

CCI, Charlson Comorbidity Index.

*Pooled estimate of five imputations. The entire study population varied between 3298 and 3318 patients.

**Participants with value G1 were added to G2 (<1% of data prior to multiple imputation). From 2004, the WHO 1999‐grading system was used, previously, the WHO 1973‐grading was used.

***Participants with missing education level were added to low (5% of data prior to multiple imputation).

****Participants with year of diagnosis 1999 or later included.

**Figure 2:**
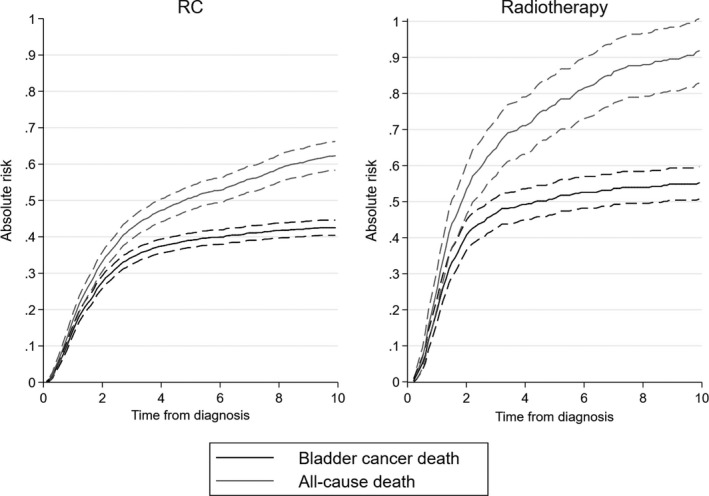
Absolute risk of death, separated by bladder cancer death and all‐cause of death for patients with metastasis‐free muscle‐invasive urothelial carcinoma treated with radical cystectomy (RC) and radiotherapy in the entire study population

Patients treated with radiotherapy, as compared to RC, had a higher risk of bladder cancer death and all‐cause death. Five years after diagnosis, the absolute risk of bladder cancer death was 51% (95% CI, 47%‐55%) for patients treated with radiotherapy, and 39% (95% CI, 37%‐41%) for RC (Figure 2). Corresponding risks for all‐cause death were 77% (95% CI, 69%‐85%) and 51% (95% CI, 47%‐54%), respectively. Relative risks of bladder cancer death and all‐cause death for radiotherapy versus RC increased with time from diagnosis. At 2 years from diagnosis, the multivariable adjusted relative risk was 1.58 for bladder cancer death and 1.54 for all‐cause death. At 5 years from diagnosis, the corresponding risks were 2.38 and 2.19, respectively (Table 2), derived from results in Table S1.

**Table 2:** Estimates of relative risk (hazard ratios) of treatment (radiotherapy versus RC) for bladder cancer death and all‐cause death in the entire study population and “trial population” using multivariate adjusted and propensity‐score matched analysis at different point in times from diagnosis. Results derived from data in Tables S1 and S4

	Time from diagnosis	Entire study population, multivariate adjusted model	“Trial population,” multivariate adjusted model	“Trial population,” propensity‐score matched model
Bladder cancer death, HR (95% CI)				
RC	—	1, ref	1, ref	1, ref
Radiotherapy	2 years	1.58 (1.12‐2.22)	1.67 (1.06‐2.64)	1.53 (0.85‐2.77)
	5 years	2.38 (1.36‐4.15)	2.40 (1.15‐4.98)	2.37 (0.90‐6.24)
	Any year	1.52 (1.31‐1.77)[Link]	1.64 (1.33‐2.00)*	1.51 (1.17‐1.94)*
All‐cause death, HR (95% CI)				
RC	—	1, ref	1, ref	1, ref
Radiotherapy	2 years	1.54 (1.20‐1.97)	1.61 (1.16‐2.22)	1.39 (0.93‐2.07)
	5 years	2.19 (1.51‐3.18)	2.12 (1.30‐3.45)	1.73 (0.96‐3.13)
	Any year	1.62 (1.43‐1.83)[Link]	1.70 (1.44‐2.01)*	1.49 (1.23‐1.81)*

Multivariate models were adjusted for age at diagnosis (continuous), calendar year at diagnosis (in two categories), education level, gender, marital status, comorbidity, health‐care region, reporting unit category, T category, systemic chemotherapy, and grade.

RC, radical cystectomy.

*Proportional hazards assumption violated.

The “trial population” included 2 154 patients from the study population matching the SPARE‐trial inclusion criteria^10^ as described above and in Figure 1. In this “trial population,” 13% were treated with radiotherapy and 87% with RC, with similar results as the entire study population, Table 2, Table S2, Table S4, and Figure S1. After exclusion of the first patient in each reporting unit, we performed an instrumental variable analysis of the 2 104 patients in the “trial population.” An analysis of the assumptions for the instrumental variable analysis is shown in Tables S3 and Table S5, and Figure S2. Results from the two‐stage least squares regression of the instrument showed an adjusted risk difference at 5 years after diagnosis—2 (95% CI, −48 to 43) events of bladder cancer death and −6 (95% CI, −41 to 29) events of all‐cause deaths, implying only a small difference between the treatment groups Table 3. These point estimates show a decreased risk for radiotherapy as compared to RC of 2 events of bladder cancer death and 6 events of all‐cause death, per 100 patients, but with large confidence intervals. For comparison, we have also calculated these risk differences using ordinary least squares regression (Table S6).

**Table 3:** Risk differences from two‐stage least squares regression based on instrumental variable analysis for bladder cancer death and all‐cause death for patients treated with radiotherapy as compared to radical cystectomy in the “trial population,” per 100 patients

Time from diagnosis	Bladder cancer death		All‐cause death	
	Unadjusted	Adjusted*	Unadjusted	Adjusted*
2 years	1 (−30 to 41)	4 (−33 to 41)	−3 (−29 to 36)	−2 (−31 to 36)
5 years	−14 (−58 to 29)	−2 (−48 to 43)	−16 (−51 to 19)	−6 (−41 to 29)

CCI, Charlson Comorbidity Index.

*Adjusted for gender, T category, grade, CCI, education level, marital status, age at diagnosis (continuous), systemic chemotherapy, and calendar time at diagnosis (in two categories).

## 4 DISCUSSION

Patients with muscle‐invasive urothelial carcinoma without metastases treated with radiotherapy as compared with RC had higher risk of bladder cancer death and all‐cause death. However, when taking patient selection into account and restricting the population to those eligible for a trial, our data implied similar survival for a group of patients with high likelihood of both treatments.

The main strengths of the current study are the sample size, the quality of the data, and the nation‐wide recruitment base. We had access to several high‐quality, nationwide registers with detailed data on tumor characteristics, treatment, and covariates such as comorbidity and education level.^11^ We used multiple imputations to account for selection mechanisms to either treatment, in combination with instrumental variable analysis to account for patient selection between the treatment groups, not detected by the covariates.^17^ The main limitations are the difference in characteristics between the treatment groups as a result of the national treatment guidelines, and an uncertainty in the received radiotherapy dose. Moreover, we have no information about received dose and treatment fields for the patients treated with radiotherapy. As we have no data of patients not receiving the full dose of external beam radiation, we have performed the study on an intention‐to‐treat basis, thus, we have not accounted for incomplete treatments. Data in the SNRUBC have not been systematically validated versus clinical journals; hence, we have no information of the proportion of misclassification in the separate variables. Moreover, regional lymph nodes were not irradiated, and pelvic lymphadenectomy was an integral part of most patients treated with RC (only 11% of the study population treated after 2008 did not undergo a lymphadenectomy). In the SNRUBC, treatment with radiotherapy was classified as curative from 2008 onwards. Before 2008, radiotherapy could also be registered as non‐curative; however, as the guidelines do not support palliative radiotherapy for this study population, we believe the number of individuals treated with palliative radiation is negligible. We tried to overcome this misclassification of the exposure by selecting patients with urothelial carcinoma with no metastases, and to perform an analysis restricted to patients that theoretically could be included in a randomized controlled trial. However, we have not been able to assess all inclusion criteria, such as hydronephrosis and the presence of widespread carcinoma in situ.^14^, ^18^

This study aimed to address survival after radiotherapy vs RC over a period of more than 15 years. During this time, the guidelines for both RC and radiotherapy have changed. The Swedish guidelines from May 2013 have recommended chemoradiotherapy over radiotherapy alone; however, during the study period chemoradiotherapy was not applied. However, these changes in guidelines have in most studies improved the survival marginally for both treatments. As these changes have not been implemented in a consistent manner, the current study has not been able to specifically account for those differences over the study period. We had no intention to investigate chemoradiotherapy,^8^, ^19^, ^20^, ^21^, ^22^, ^23^ which has been reported to provide improved survival in comparison to radiotherapy, because this combined treatment‐modality was not used in Sweden during the study period. Furthermore, some patients might be unsuitable for concurrent chemotherapy due to specific comorbidities, and were subsequently treated with radiotherapy as the second best bladder‐sparing option. Moreover, we did not categorize patients differently depending on perioperative chemotherapy given in association with RC, as the majority of patients treated with RC did not receive any of these treatments, and we had no intention to investigate subgroups where RC or radiotherapy would be more beneficial. The absence of information about clinically prognostic factors such as extent of transurethral resection prior to radiotherapy, performance status, and information on presence of hydronephrosis prior to treatment were not available. Thus, adjustment for these factors was not possible in the present study.

The optimal design for the current research question is a prospective randomized controlled trial. However, the largest trial to date investigating radiotherapy vs RC in terms of overall survival requiring a sample size of 1 015 patients was closed prematurely due to poor accrual.^9^, ^14^, ^18^ Another trial from the 1980s based on 189 patients, found no statistically significant difference in overall survival, but indicated that the prognosis was influenced by age, gender, treatment responses, and tumor characteristics.^22^ In the absence of randomized results, real‐world results from population‐based observational studies can provide useful complementary data with high validity,^14^, ^23^ despite the restrictions mentioned above.

Similar to most previous observational reports, baseline data in our study showed substantial differences in age and comorbidity between the treatments.^24^, ^25^ Previous studies show 5‐year overall survival for radiotherapy ranging from 21% to 30% and for RC from 36% to 47%,^24^, ^25^ and most relative risk estimates of all‐cause death using standard multivariable adjustments are in the range of 1.4‐1.5,^25^, ^26^ with an exception of a recent study with an estimate of 2.^27^ The relative risk estimates in our study increased with time from diagnosis. Previous studies have reported evidence of nonproportional hazards over time. For example, a very recent comparison of RC to chemoradiotherapy reported a relative risk decrease over time using chemoradiotherapy as reference group,^28^ and has in another study not been investigated further.^26^

We accounted for patient selection between the treatments by use of an instrumental variable analysis. When applying this method, the results showed similar survival between the treatments, but with a wide confidence interval. Given that the instrumental variable assumptions are valid, the analysis is expected to provide an estimate closer to the true treatment effect than relative risk estimates.^29^, ^30^ We have used the recommended guidelines for reporting instrumental variable analysis;^31^ however, as some of the underlying assumptions cannot be empirically verified,^17^ there is always a possibility that our analysis might violate some of these assumptions.^32^

A large Canadian cohort study of 5 259 patients reported no differences in cancer‐specific survival for patients treated with radiotherapy as compared to RC after propensity score matching, and patients treated with radiotherapy had slightly higher overall survival after 5 years.^24^ Other large studies using instrumental variable analysis have reported inconsistent results: both no survival differences^26^ and better survival for patients treated with RC.^25^

Much like other international settings, the Swedish national guidelines recommends radiotherapy to patients with high‐surgical risk. This treatment selection may have therefore hampered the possibility to accurately evaluate radiotherapy as an alternative to RC in a retrospective manner. The unadjusted absolute risk estimates and the relative risk estimates can be generalized to populations of patients with similar background characteristics, treatment and progression patterns as the current study. In contrast, estimates from the instrumental variable analysis reflect results in those patients in whom the treatment choice depends on the instrument, that is, to the patients who would have been treated differently depending on treatment preferences in the units.^26^, ^33^ We speculate that this group of patients are more often older and/or frail, as compared to those that would be offered RC regardless of treating unit.

In conclusion, patient selection mechanisms make it difficult to evaluate results from conventionally adjusted or propensity‐score matched survival analysis comparing RC and radiotherapy. When taking into account unmeasured confounding by instrumental variable analysis, similar survival for the treatments were found for a group of patients which we speculate to include a selection of older and/or frail patients in the “trial population.” Further observational studies are needed to characterize this group with similar survival regardless of treatment, which can serve as a basis for future comparisons with respect to modern chemoradiotherapy.

## CONFLICT OF INTEREST

None declared.
