# Supplementary material for: Survival after radiotherapy versus radical cystectomy for primary muscle‐invasive bladder cancer: A Swedish nationwide population‐based cohort study
Source: Cancer Med. 2019 Apr 1;8(5):2196–204. doi: 10.1002/cam4.2126 (PMC6536982; doi:10.1002/cam4.2126)
Supplement: Supplementary file 1 [file CAM4-8-2196-s001.docx]

**Supplementary Material**

**Methods S1**

In the analysis of the “trial population” (selected according to SPARE trial eligibly criteria,^1^ i.e. 60 to 80 years of age at diagnosis with T category 2 or 3 and CCI less than 3), we further calculated relative risks after propensity score matching for covariates and risk difference using instrumental variable analysis to further account for patient selection between the treatment groups.^2^

Data from RC treatment were matched on data from radiotherapy, via propensity scores. Specifically, a logistic propensity score model of the type of treatment received was created, and the following variables were included in the model: age at diagnosis (continuous), calendar year at diagnosis (in two categories), education level, gender, marital status, comorbidity, systemic chemotherapy, T category, and grade. Matching across the two groups was achieved with a 0.1-width caliper with no replacement and the balancing property was satisfied.

Risk difference was calculated by use of treatment preference for patients treated previously at the same reporting unit as an instrument in two-stage least squares (2SLS) regression analysis.^3^ The instrument was quantified by the proportion (0 to 1) of RCs among previous patients at the same unit, whilst excluding the first patient in each unit from the analysis. This choice of instrumental variable was based on the following assumptions: 1) The proportion of RCs among the previous patients at the same unit is associated with the actual treatment for the current patient, 2) The proportion of RCs among the previous patients at the same unit does not affect the risk of bladder cancer or all-cause death for the current patient other than through the actual treatment, 3) The proportion of RCs among the previous patients at the same unit is not associated with known and unknown covariates affecting the risk of bladder cancer or all-cause death for the current patient. These assumptions were checked by tabulating the instrumental variable in tertiles versus the mean proportion of RC among previous and actual treatments, covariates, and events of bladder cancer and all-cause deaths.

The two-stage least squares (2SLS) regression analysis were calculated unadjusted, and adjusted for gender, T category, grade, systemic chemotherapy, CCI, education level, marital status, age at diagnosis (continuous) and calendar year at diagnosis (1997-2005, and 2006-2014). The results from the 2SLS regression based on the instrument were compared with results for ordinal least square (OLS) regression based on the actual treatment at 2, and 5 years after diagnosis. The strength of the instrument was determined with results from first stage regression in the 2SLS analysis.

The OLS regression of actual treatment showed an increased risk for patients treated with radiotherapy as compared to RC, **Table S6**; the adjusted risk difference at 5 years after diagnosis was 20 (95% CI, 11-28) events of bladder cancer death and 18 (95% CI, 10-25) of all-cause deaths per 100 patients. First stage results for each of the imputed dataset resulted in F-statistics from 50 to 90 and p-values for F-test of excluded instruments <0.0001, separately.

**References**

1 Huddart RA, Birtle A, Maynard L, Beresford M, Blazeby J, Donovan J, et al. Clinical and patient-reported outcomes of SPARE - a randomised feasibility study of selective bladder preservation versus radical cystectomy. Bju Int. 2017;120(5):639-50.

2 Agoritsas T, Merglen A, Shah ND, et al. Adjusted Analyses in Studies Addressing Therapy and Harm: Users' Guides to the Medical Literature. JAMA. 2017;317(7):748-759.

3 Baum, C. F. ivreg2: Stata module for extended instrumental variables/2SLS, GMM and AC/HAC, LIML and k-class regression. http://ideas.repec org/c/boc/bocode/s425401.html. 2007.

**Supplementary Tables and Figures**

**Table S1.** Detailed information on relative risk estimates for radiotherapy vs RC in multivariate Cox models with treatment as time-by-covariate interaction models in the entire study population.

| **Treatment terms in the models** | **Cox model with no time varying covariates** | **Cox model with time-by-covariate interactions** |
| --- | --- | --- |
|  | **HR (95% CI)** | **HR (95% CI)** |
| **Bladder cancer death** | | |
| Treatment (RT vs RC) | 1.52 (1.31 - 1.77) | 1.20 (0.98 - 1.47) |
| Treatment* Time |  | 1.15 (1.07 - 1.23) |
| **All-cause death** | | |
| Treatment (RT vs RC) | 1.62 (1.43 - 1.83) | 1.22 (1.03 - 1.43) |
| Treatment* Time |  | 1.12 (1.08- 1.17) |

Cox models were adjusted for age at diagnosis (continuous), calendar year at diagnosis (in two categories), education level, gender, marital status, comorbidity, health care region, reporting unit category, systemic chemotherapy, T category, grade, M category and N category.

**Table S2.** Baseline characteristics of “trial population” with respect to actual treatment.

| **Covariate** | **Categories** | **Radical Cystectomy**  **N=1874*** | **Radiotherapy**  **N=280*** |
| --- | --- | --- | --- |
|  |  |  |  |
| **Gender** | **Men** | 76% | 78% |
|  | **Women** | 24% | 22% |
|  |  |  |  |
| **Age** | **Below 75 years** | 77% | 45% |
|  | **75 years and above** | 23% | 55% |
|  |  |  |  |
| **Calendar year of diagnosis** | **1997-2005** | 41% | 55% |
|  | **2006-2014** | 59% | 45% |
|  |  |  |  |
| **T category** | **T2** | 81% | 81% |
|  | **T3** | 19% | 19% |
|  |  |  |  |
| **Grade** | **G2** | 11% | 12% |
|  | **G3** | 89% | 88% |
|  |  |  |  |
| **CCI** | **0** | 73% | 52% |
|  | **1** | 15% | 30% |
|  | **2** | 11% | 18% |
|  |  |  |  |
| **Education level** | **Low** | 47% | 53% |
|  | **Medium** | 36% | 33% |
|  | **High** | 17% | 14% |
|  |  |  |  |
| **Maritial status** | **Married** | 62% | 61% |
|  | **Divorced/Widowed** | 28% | 30% |
|  | **Unmarried** | 10% | 9% |

*Pooled estimate of five imputations. The “trial population” varied between 2144 and 2166 patients.

**Table S3.** Baseline characteristics of “trial population” with respect to actual treatment, A) matched for propensity scores and B) in tertiles of the instrumental variable.

|  |  | **A** | | **B** | | |
| --- | --- | --- | --- | --- | --- | --- |
| **Covariate** | **Categories** | **Propensity score matching analysis** | | **Instrumental variable analysis** | | |
|  |  | **RC**  **N=273*** | **Radiotherapy**  **N=273*** | **Tertile 1**  **N=706**** | **Tertile 2**  **N=703**** | **Tertile 3**  **N=695**** |
| **Mean proportion of RC among previous treatments** |  | - | - | 99% | 94% | 81% |
| **Mean actual treatment RC** |  | - | - | 93% | 90% | 79% |
|  |  |  |  |  |  |  |
| **Gender** | **Men** | 78% | 77% | 78% | 75% | 76% |
|  | **Women** | 22% | 23% | 22% | 25% | 24% |
|  |  |  |  |  |  |  |
| **Age** | **Below 75 years** | 36% | 48% | 72% | 72% | 75% |
|  | **75 years and above** | 64% | 52% | 28% | 28% | 25% |
|  |  |  |  |  |  |  |
| **Calendar year of diagnosis** | **1997-2005** | 62% | 54% | 48% | 30% | 46% |
|  | **2006-2014** | 38% | 46% | 52% | 70% | 54% |
|  |  |  |  |  |  |  |
| **T category** | **T2** | 84% | 81% | 80% | 86% | 80% |
|  | **T3** | 16% | 19% | 20% | 14% | 20% |
|  |  |  |  |  |  |  |
| **Grade** | **G2** | 14% | 12% | 13% | 11% | 10% |
|  | **G3** | 86% | 88% | 87% | 89% | 90% |
|  |  |  |  |  |  |  |
| **CCI** | **0** | 36% | 53% | 70% | 68% | 72% |
|  | **1** | 38% | 29% | 16% | 18% | 18% |
|  | **2** | 26% | 18% | 13% | 14% | 10% |
|  |  |  |  |  |  |  |
| **Education level** | **Low** | 57% | 53% | 49% | 43% | 51% |
|  | **Medium** | 29% | 33% | 36% | 38% | 35% |
|  | **High** | 14% | 15% | 16% | 19% | 15% |
|  |  |  |  |  |  |  |
| **Maritial status** | **Married** | 61% | 62% | 62% | 60% | 63% |
|  | **Divorced/**  **Widowed** | 31% | 30% | 29% | 30% | 26% |
|  | **Unmarried** | 8% | 9% | 8% | 10% | 11% |

*Pooled estimate of five imputations. The propensity score analysis based on the “trial population” varied between 266 and 278 patients

**Pooled estimate of five imputations. The instrumental variable analysis based on the “trial population” varied between 2096 and 2117 patients, as the first patient in each unit was excluded.

**Table S4**. Detailed information on relative risk estimates for radiotherapy vs RC in Cox models with treatment as time-by-covariate interaction models in multivariate adjusted and propensity score matched analysis in the “trial population”.

| **Treatment terms in the models** | **Cox model with no time varying covariates** | | **Cox model with time-by-covariate interactions** | |
| --- | --- | --- | --- | --- |
|  | **Multivariate adjusted model** | **Propensity score matched model** | **Multivariate adjusted model** | **Propensity score matched model** |
|  | **HR (95% CI)** | **HR (95% CI)** | **HR (95% CI)** | **HR (95% CI)** |
| **Bladder cancer death** | | | | |
| Treatment (RT vs RC) | 1.64(1.33–2.00) | 1.51 (1.17-1.94) | 1.32 (1.00 – 1.73) | 1.15 (0.81-1.62) |
| Treatment* Time |  |  | 1.13 (1.03 - 1.24) | 1.16 (1.02-1.13) |
| **All-cause death** | | | | |
| Treatment (RT vs RC) | 1.70 (1.44–2.01) | 1.49 (1.23-1.81) | 1.34 (1.08 - 1.66) | 1.20 (0.92-1.57) |
| Treatment* Time |  |  | 1.10 (1.04 - 1.16) | 1.08 (1.01-1.15) |

Multivariate adjusted model were adjusted for age at diagnosis (continuous), calendar year at diagnosis (in two categories), education level, gender, marital status, comorbidity, health care region, reporting unit category, systemic chemotherapy, T category, and grade.

**Table S5**. Distribution of endpoints in tertiles of the instrumental variable based on the “trial population”.

| **Endpoint** | **Timing of event** | **Number of events** | **Instrumental variable (%)** | | |
| --- | --- | --- | --- | --- | --- |
|  |  |  | **Tertile 1** | **Tertile 2** | **Tertile 3** |
| **Bladder cancer death*** | **2 years** | **No event** | 63% | 56% | 62% |
|  |  | **Event** | 37% | 44% | 38% |
|  |  |  |  |  |  |
|  | **5 years** | **No event** | 50% | 46% | 48% |
|  |  | **Event** | 50% | 54% | 52% |
|  |  |  |  |  |  |
|  |  |  |  |  |  |
| **All-cause death** | **2 years** | **No event** | 63% | 56% | 62% |
|  |  | **Event** | 37% | 44% | 38% |
|  |  |  |  |  |  |
|  | **5 years** | **No event** | 50% | 46% | 48% |
|  |  | **Event** | 50% | 54% | 52% |

* Death from other causes within the time period handled as censored

|  | **Bladder cancer death** | | **All-cause death** | |
| --- | --- | --- | --- | --- |
| **Timing of event** | **Unadjusted** | **Adjusted*** | **Unadjusted** | **Adjusted*** |
| **Ordinary Least Squares (actual treatment)** | | | | |
| **2 years** | 20 (13 - 27) | 16 (9 - 23) | 21 (15 - 27) | 16 (10 - 23) |
| **5 years** | 22 (13 - 30) | 20 (11 - 28) | 22 (15 - 29) | 18 (10 - 25) |

**Table S6.** Risk differences by ordinary least squares for **bladder cancer death** and **all-cause death** for patients treated with radiotherapy as compared to radical cystectomy in the “trial population”, per 100 patients.

**
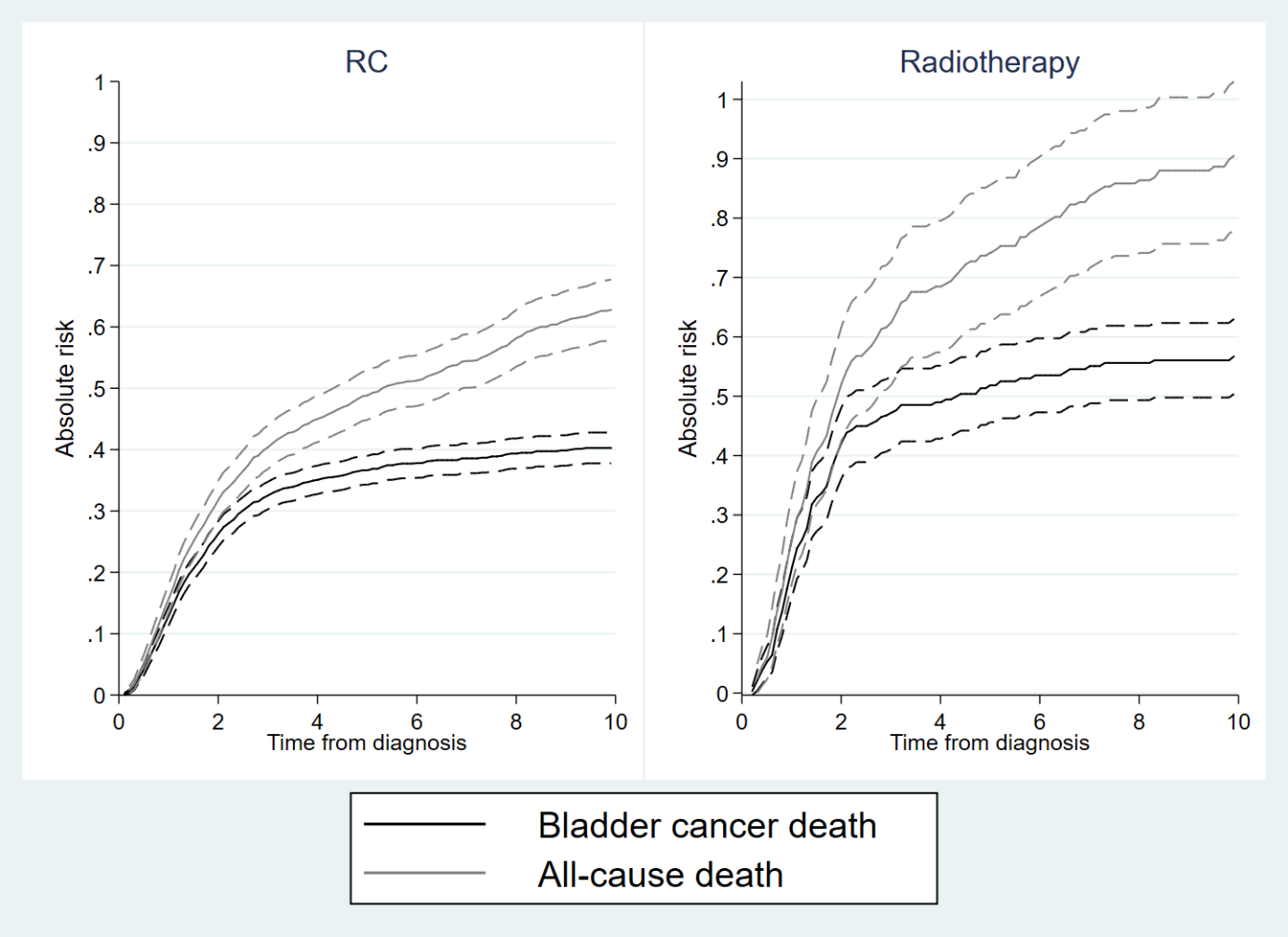
**

**Figure S1**. Absolute risk of death, separated by bladder cancer death and other causes of death in the “trial population”.

**
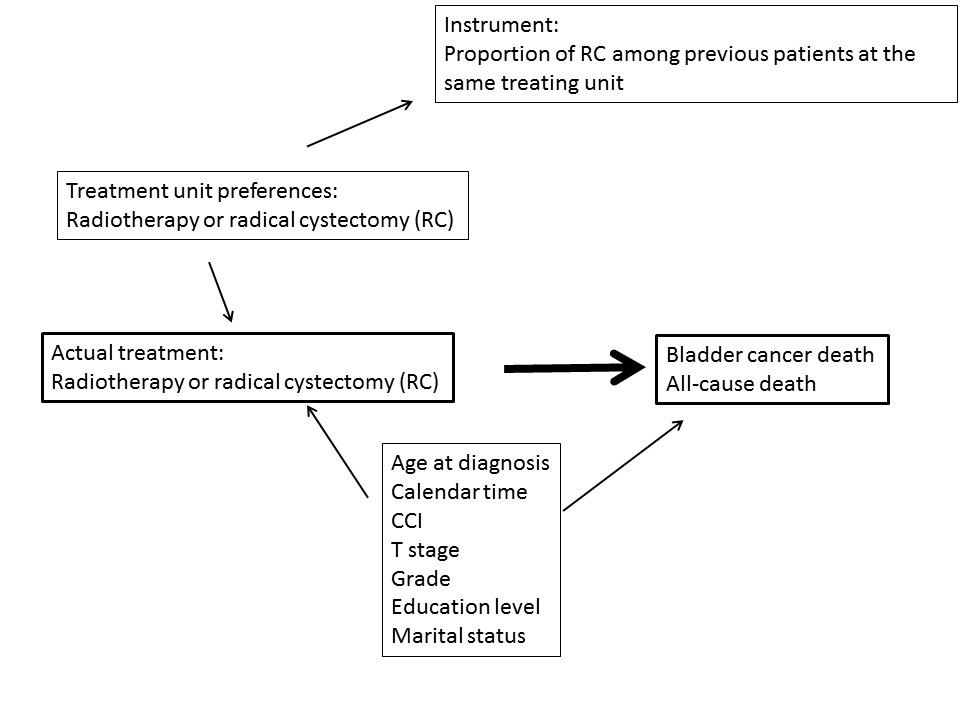
Figure S2**. Directed acyclic graph (DAG) of associations for the instrumental variable analysis. The bold arrow represent the association investigated in the current study.
